# The epidemiology of canyoning rescue operations in Italy: a retrospective 15-year database study

**DOI:** 10.1186/s13049-025-01535-8

**Published:** 2026-01-15

**Authors:** Alexandre Tomasi, Michiel Jan Van Veelen, Marco Biasioni, Tomas Dal Cappello, Giacomo Strapazzon

**Affiliations:** 1 Institute of Mountain Emergency Medicine, Eurac Research, Bolzano, Italy; 2 Corpo Nazionale Soccorso Alpino e Speleologico - CNSAS, Milano, Italy; 3 Department of Sport Science, Medical Section, University of Innsbruck, Innsbruck, Austria; 4 Department of Medicine – DIMED, University of Padova, Padova, Italy; 5 Italian Society of Mountain Medicine – SIMeM, Padova, Italy

**Keywords:** Canyoning, Wilderness, Search and rescue, Trauma, Epidemiology

## Abstract

**Background:**

Canyoning has become increasingly popular in recent years and led to an increase in rescue operations between 15 and 40% in specific areas. We aimed to study the epidemiology and characteristics of canyoning rescue operations (CRO) in Italy.

**Methods:**

We retrospectively collected data from all reports of CRO carried out in Italy for 15 years (from 2009 to 2023) from a nationwide database of the Corpo Nazionale Soccorso Alpino e Speleologico (CNSAS). Data included event details, patient demographics, injury type and severity, and evacuation methods. Descriptive and inferential statistical analyses were performed, with a significance level set at *p*<0.05.

**Results:**

A total of 344 CROs involving 569 patients were analysed. Trauma accounted for 45% of cases, with lower extremity injuries being the most common (42%). Medical emergencies represented 8% of cases, while 263 (46%) patients were uninjured. Severe injuries or illnesses were observed in 9 patients (3%), and 39 patients (13%) died, primarily due to drowning and trauma. The median rescue time was 160 (100-270) min. Helicopters were used in 68% of hospital transports, particularly in more severe cases. CRO times of accidents supported by helicopter were not different from accidents supported by ambulance.

**Conclusions:**

CRO were seasonally related, with an increasing trend over the years. Accidents typically resulted in minor injuries but patients were exposed, despite HEMS support, for long time to environmental factors. Polytrauma and drowning were the leading cause of fatalities.

## Background

Canyoning is a recreational activity that has grown rapidly in recent years, with rescue operations increasing by 15 to 40% in some regions [1]. It involves several inherent risks related both to the activity and to the environment. One major hazard is flash flooding caused by sudden heavy rainfall or the release of water from upstream dams. Canyoners caught in a flash flood may suffer major trauma or drowning [2, 3]. Slips and falls on wet, unstable terrain are common and may result in fractures, dislocations, or head injuries. Accidental hypothermia is another key concern. Immersion in water increases heat loss by roughly twenty-five-fold, particularly in flowing water, where temperatures are often below 25 °C and currents are turbulent [4]. Prolonged exposure to cold water, even when wearing a wetsuit, can cause a dangerous drop in core temperature and impair cognitive and physical performance [5, 6], increasing the risk of errors, injury, or death.

Canyoning sites are typically remote, with limited mobile or GPS coverage, which delays emergency calls. Rescue teams often deploy late in the day or at night [2, 3], and the interval from accident to evacuation frequently exceeds 60 min [3, 7].

Canyoning rescue requires specialised skills, including safe movement on slippery terrain, ropework, and swimming in whitewater, as well as appropriate protective equipment to minimise the risk of hypothermia and drowning [2]. Ground operations may involve rappelling down waterfalls or steep rock, requiring specific technical training. Helicopter evacuations are also challenging, often involving winch operations and other complex manoeuvres [3].

The Medical Commission of the International Commission for Alpine Rescue (ICAR MedCom) has issued consensus guidelines for on-site management and transport of patients during canyoning rescue operations (CRO), based on the best available evidence [7]. However, current recommendations still rely heavily on expert opinion due to limited data.

The aim of this study is to describe the epidemiology and characteristics of canyoning accidents, type and severity of injuries or illness, and the associated rescue operations, using data of CRO recorded in a nationwide database from 2009 and 2023.

## Methods

### Data

Data from CRO recorded in the Corpo Nazionale Soccorso Alpino e Speleologico (CNSAS) database were collected retrospectively.

### Variables

We accessed the anonymised dataset and extracted the following variables: time of the event (day and hour); season; location; age and gender; mechanism, location, and severity of the injury or illness; type of evacuation (ground or air); evacuation duration; and number of rescuers involved. Rescuers specialised in canyoning rescue receive training comparable to that of an Emergency Medical Responder (EMR). Their competencies include basic cardiopulmonary resuscitation with semi-automated defibrillation, basic airway management, haemorrhage control, and initial trauma management according to Basic Trauma Life Support (BTLS) principles. They are not authorised to administer medications. CNSAS medical personnel are physicians and nurses trained in Advanced Cardiovascular Life Support (ACLS) and certified in trauma management according to Prehospital Trauma Life Support (PHTLS) standards. We report descriptive statistics of the accidents. Patients were grouped by age into paediatric (<16 years), young adult (16–40 years), adult (41–64 years) and elderly (>64 years). Rescue times were divided into two daytime intervals (9am-3pm and 3pm-9pm) and one nighttime interval (9pm-9am). As canyoning is commonly practiced during daylight hours, the 12-hour period was split into two equal segments. Injury patterns were classified as uninjured, medical or environmental, trauma, search or not found. The injury severity scoring system used by CNSAS consists of 5 codes, which are a simplified form of the National Advisory Committee for Aeronautics (NACA) score [8–10] (Table 1).

**Table 1:** Patient severity codes

Code	Patient condition
0	Uninjured
1	Minor injury with stable vital signs
2	Moderate injury with risk of instability
3	Severe and potentially life-threatening injury
4	Dead
5	Patient not found/Unsuccessful search and rescue

### Statistical analysis

SPSS version 29 (IBM Corp., Armonk, NY, USA) was used for statistical analysis. Pearson correlation was performed to assess a trend for the number of accidents during the period 2009-2023. For continuous variables Kruskal-Wallis test was used to compare more than two groups and Mann-Whitney U test to compare two groups. For multiple comparisons *p*-values were corrected by means of Holm-Bonferroni method. Fisher’s exact test was performed to compare two proportions. Normal distribution was assessed by means of Shapiro-Wilk test and normal Q-Q plots. Data were reported as mean ± standard deviation if they were normally distributed, otherwise as median (interquartile range). *P*<0.05 (two-sided) was considered as statistically significant.

### Ethical approval

The ethical committee of the Bolzano Hospital approved the study (No. 0038423).

## Results

During the 15-year study period, a total of 344 CROs were conducted. Among 569 patients, 137 (24%) were female; gender was unknown for three patients. The mean age was 39±15 years, with 90% of patients aged 16–64 years. The remaining 10% was equally distributed between those younger than 16 and those older than 64 years. Most patients were from Italy (79%), followed by France (5%), Belgium (3%), Germany (2%), Netherlands (2%), USA (1%) and other countries (8%).

### Type and severity of injuries or illnesses

Traumatic injuries were recorded in 253 patients (45%), while 46 (8%) experienced medical conditions. A total of 263 patients (46%) were uninjured, and seven (1%) were not found (Table 2). Lower extremity injuries were the most frequent (42%), followed by polytrauma (12%), upper extremity injuries (9%), accidental hypothermia (7%), spine injuries (6%), and drowning (5%). The severity distribution of injuries and illnesses is shown in Table 3, with severe cases identified in nine patients (3%). Fatalities at the scene involved 39 patients (13%), mostly due to traumatic injuries (20 patients) and drowning (16 patients). Suicide accounted for eight traumatic deaths (21%). Moderate to severe injuries or deaths were more common among medical cases than traumatic ones (57% vs. 26%, *p*<0.001).

**Table 2:** Type of injury and illness in patients of canyoning rescue operations. Years 2009-2023

**Injury/illness**	***n***	**%**	**Type of injury/illness**	***n***	**%**
Traumatic injury	253	44.5%	Head/face	9	3.0%
			Spine/back	19	6.4%
			Thorax	7	2.3%
			Upper extremity	27	9.0%
			Lower extremity	127	42.5%
			Pelvis	11	3.7%
			Polytrauma	37	12.4%
			Minor multiple injuries	10	3.3%
			Unknown	6	2.0%
Medical/environmental illness	46	8.1%	Hypothermia	20	6.7%
			(Near) drowning	16	5.4%
			Medical illness ^a^	10	3.3%
			Total injuries and illnesses	299	100.0%
Victim not found	7	1.2%			
Uninjured	263	46.2%			
Total	569	100.0%			

^a^ snake bite (*n*=1), hypoglycaemia (*n*=1), seizures (*n*=1), malaise (*n*=2), sickness (*n*=4), dehydration/exhaustion (*n*=1)

**Table 3:** Injury and illness by severity code. Uninjured (code 0, *n*=263) and not found patients (code 5, *n*=7) are not shown. Years 2009-2023

Severity	Traumatic injury		Medical/		Total	
			Environmental			
			Illness			
	*n*	%	*n*	%	*n*	%
Minor-moderate (code 1–2)	227	89.7%	24	52.2%	251	83.9%
Severe (code 3)	6	2.4%	3	6.5%	9	3.0%
Lethal (code 4)	20	7.9%	19	41.3%	39	13.0%
Total	253	100.0%	46	100.0%	299	100.0%

### Characteristics and time of CROs

**Fig. 1:**
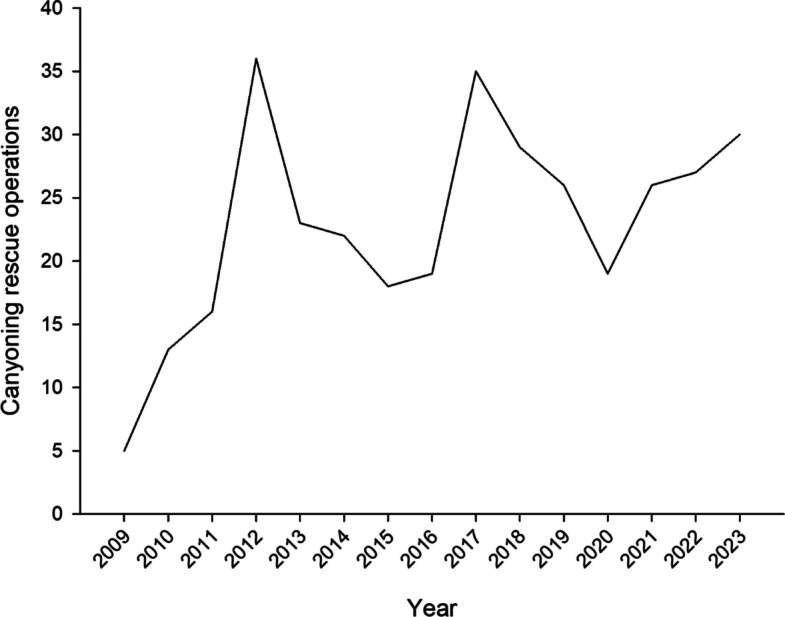
Total canyoning rescue operations per year during the period 2009-2023

**Fig. 2:**
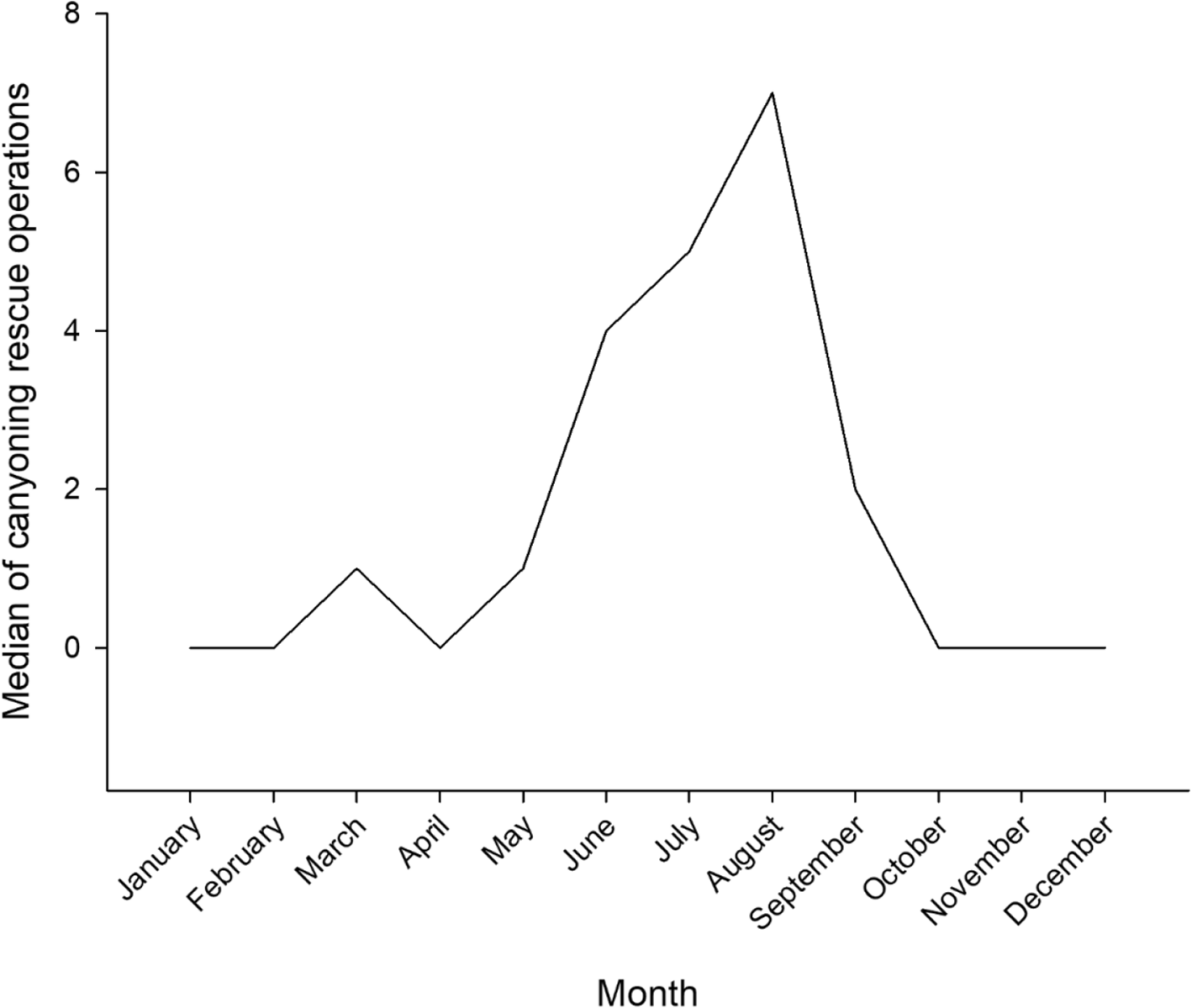
Median number of canyoning rescue operations (CRO) per month. Years 2009-2023

The mean annual number of CROs was 23±8, showing an increasing trend from 2009 to 2023 (*r*=0.541, *p*=0.037) (Fig. 1). Most accidents (72%) occurred on weekends, national holidays and in August, while 28% occurred during the rest of the year. Monthly accident frequency varied significantly (*p*<0.001), increasing in late spring and reaching a maximum in August (Fig. 2). Most patients (82%) were uninjured or mildly injured (Table 4). Excluding “no return” alarms (whose patients were uninjured in 97% of cases and with only minor injuries in 3% of cases), falls and slips were the leading causes of accidents, resulting in moderate to severe injuries in 23% and 14% of cases, respectively (Table 4). Emergency calls were more frequent during daytime (93%; 51% between 9 am and 3 pm and 42% between 3 pm and 9 pm) than nighttime (7%, 9 pm-9 am). Excluding CROs in which no victim was found, in daytime 60% of patients were injured or ill, compared to 19% at night (*p*<0.001).

**Table 4:** Accident cause by severity code. Years 2009-2023

**Cause**	**Severity code**												**Total**
	**0**		**1**		**2**		**3**		**4**		**5**		
	***n***	**%**	***n***	**%**	***n***	**%**	***n***	**%**	***n***	**%**	***n***	**%**	
Bad weather	16	100.0%	0	0.0%	0	0.0%	0	0.0%	0	0.0%	0	0.0%	16
Companion	15	100.0%	0	0.0%	0	0.0%	0	0.0%	0	0.0%	0	0.0%	15
No return	143	97.3%	4	2.7%	0	0.0%	0	0.0%	0	0.0%	0	0.0%	147
Equipment	0	0.0%	0	0.0%	2	100.0%	0	0.0%	0	0.0%	0	0.0%	2
Fall from height	9	6.3%	96	67.6%	29	20.4%	4	2.8%	4	2.8%	0	0.0%	142
Fatigue	18	81.8%	4	18.2%	0	0.0%	0	0.0%	0	0.0%	0	0.0%	22
Flood	1	5.0%	9	45.0%	0	0.0%	0	0.0%	10	50.0%	0	0.0%	20
Incapacity	53	86.9%	8	13.1%	0	0.0%	0	0.0%	0	0.0%	0	0.0%	61
Loss of orientation	4	100.0%	0	0.0%	0	0.0%	0	0.0%	0	0.0%	0	0.0%	4
Rappelling	0	0.0%	2	66.7%	0	0.0%	0	0.0%	1	33.3%	0	0.0%	3
Search	0	0.0%	0	0.0%	0	0.0%	0	0.0%	0	0.0%	7	100.0%	7
Road accident	0	0.0%	0	0.0%	0	0.0%	1	20.0%	4	80.0%	0	0.0%	5
Slip	4	3.9%	80	78.4%	12	11.8%	2	2.0%	4	3.9%	0	0.0%	102
Sudden illness	0	0.0%	3	30.0%	2	20.0%	2	20.0%	3	30.0%	0	0.0%	10
Suicide	0	0.0%	0	0.0%	0	0.0%	0	0.0%	8	100.0%	0	0.0%	8
Unknown	0	0.0%	0	0.0%	0	0.0%	0	0.0%	5	100.0%	0	0.0%	5
Total	263	46.2%	206	36.2%	45	7.9%	9	1.6%	39	6.9%	7	1.2%	569

The median number of patients in each CRO was 1 (1-1) with a maximum of 19. The median number of rescuers involved in a CRO was 7 (4–11). The number differed by time of the day [morning: 6 (3–9); afternoon: 7 (5–12); night: 9 (7–15); *p*<0.001], with fewer rescuers involved in morning CROs compared to afternoon (*p*<0.001) and night (*p*=0.005). Median CRO duration was 160 min (100–270) and varied according to the severity code (*p*<0.001), with longer times for code 5 compared to each of the other codes (code 0: *p*=0.002; code 1: *p*=0.011; code 2: *p*=0.035; code 3: *p*=0.030; code 4: *p*=0.042). A total of 226 canyoners (40%) were transported to the hospital. Helicopter was the most common transport mode (68%), either by direct winch or after ground transfer, followed by ambulance (31%) and private vehicles (1%). Helicopter use increased with severity: 63% of mildly injured, 75% of moderately and 100% of severely injured/ill patients. CRO duration did not differ significantly between helicopter and ambulance transport [150 (100–240) min vs. 180 (90–300) min, *p*=0.362].

## Discussion

Our fifteen-year nationwide analysis provides an overview of the frequency and characteristics of canyoning rescue operations. CRO are related to season and time. Most rescued patients were injured and remained exposed to environmental factors for a prolonged time because of delayed emergency calls and the long rescue operations, increasing the risk of clinical deterioration. Ground rescue supported by air rescue was the most common rescue strategy, yet this combination did not shorten rescue times, which remained well above 60 min. CROs usually required more than five rescuers that are exposed to a higher risk of occupational injuries than other pre-hospital EMS personnel [11].

Canyoning accidents occurred mainly in late spring or summer. This seasonal pattern has been described previously [3, 12] and mirrors trends seen in other mountain activities, driven by increasing numbers of visitors, including tourists, hikers and extreme-sport recreationists. Most injuries were minor and involved the extremities, especially the lower limbs, reflecting movement over wet and slippery terrain, sometimes with inadequate footwear [3, 12]. Despite their low severity, prolonged exposure to a remote and harsh environment increases the risk of accidental hypothermia and clinical deterioration like in other accidents in the mountains [13]. Other common injuries include polytrauma, upper limb and spinal trauma. With trauma accounting for 85% of all medical conditions, out-of-hospital trauma management, including splinting of fractures and spinal immobilisation, must be among the core competencies of a canyon rescuer, as recommended by the ICAR MedCom guidelines [7].

Unstable or life-threatening conditions affected about 9% of all patients, but 18% of the injured ones. Medical and environmental events were less frequent (8% of the total, 15% of the injured), but often moderate to severe and life-threatening, accounting for 41% of deaths. Drowning was the leading cause of traumatic death, after exclusion of suicide. All drowning patients in our dataset died, whereas during flooding events less than 1% were uninjured and 50% died. Because medical personnel often arrives too late to influence outcomes, training canyoners - both recreational participants and non-medical rescuers - in self-rescue and resuscitation is crucial, as survival depends heavily on submersion time and early life support [14, 15]. Almost half of the patients were uninjured but still required assistance with evacuation.

During the day the proportion of injured patients was higher, while at night most patients had non-medical issues, such as incapacitation or delay, with only 3% sustaining moderate to severe injuries. Incapacitated or stranded people accounted for 19% of all calls and 38% of all patients, a considerable burden on rescue resources. In this subgroup, 93% were uninjured, while in the remaining 7% the primary injury recorded by the rescuers was accidental hypothermia. It should be noted that rescuers did not usually measure core temperature, but suspected hypothermia based on clinical assessment. Night calls may result not only from misjudging the canyon’s difficulty but also from carrying inadequate self-rescue equipment, which may be insufficient during a prolonged stay in the canyon [16]. Accidental hypothermia can occur even in minor or non-injured patients exposed to the canyon environment for longer than expected [17], and can develop within thirty minutes even when wearing a neoprene wetsuit, especially in whitewater [7]. Trauma-related immobility accelerates heat loss. Therefore, it is possible that accidental hypothermia in the canyon environment may be under-diagnosed because it is under-assessed, as it is in the urban and suburban pre-hospital settings [18, 19]. Because hypothermia, coagulopathy and acidosis worsen the trauma outcomes [20], priority should be placed on preventing further heat loss [21] and measuring body temperature whenever possible [20, 22]. ICAR MedCom guidelines recommend protecting the patient by a vapor barrier with a dry, low-conductivity, whole-body insulation, and to use active rewarming devices if possible [7].

Ground rescue supported by air rescue was the most common strategy. Most Italian helicopter EMS (HEMS) performing search and rescue operations are equipped with a winch, as recommended by ICAR MedCom [7]. These missions are often performed in challenging terrain, with limited resources and monitoring capabilities. Our data support the need to have clinically and logistically experienced HEMS personnel, specifically trained for winch operation in difficult environment [23]. Hospital arrival times were similar for helicopter and ambulance transports. Unfortunately, we could not determine whether helicopters acted primarily as support for ground teams or as the sole vehicle of rescue. All severity code 3 (severely injured or ill) patients were evacuated by helicopter, likely due to availability of advanced HEMS personnel (i.e., a physician) on board, yet intervention times still exceeded 60 min and required usually more than five rescuers.

Suicide was a major cause of death in our dataset and was determined as cause of death by the rescuers. Of note is that these CROs occurred involving persons in a canyon environment, not participating in canyoning.

### Limitations

Our study has some limitations. First, it is a database-based study, which carries potential limitations regarding the reliability of data entry. Variability among rescuers in recording observations may lead to inter-observer discrepancies, potentially impacting the consistency and accuracy of the data collected. Second, we have no follow up data on the medical treatment of the injured and ill patients. Third, the severity codes used by Italian CNSAS may be less discriminative than the NACA score and consequently may limit comparison with other studies. Finally, we have no data on how HEMS was deployed, either alone or in support of ground teams.

## Conclusions

CRO were seasonally related, with an increasing trend over the years. Accidents typically resulted in minor injuries but patients were exposed, despite HEMS support, for long time to environmental factors. Polytrauma and drowning were the leading cause of fatalities. Missions that extended overnight were generally due to incapacitation or technical difficulties but required extended rescue personnel. These findings could guide evidence-based optimization of rescue planning, operational practice, and risk-reduction measures.

## Data Availability

The dataset is available from the authors upon reasonable request, but the availability also depends on permission from the CNSAS.

## Abbreviations

CNSAS: Corpo Nazionale Soccorso Alpino e Speleologico
CRO: Canyon Rescue Operation
EMS: Emergency Medical Service
HEMS: Helicopter Emergency Medical Service
ICAR MedCom: Medical Commission of the International Commission for Alpine Rescue
NACA: National Advisory Committee for Aeronautics
SAR: Search and Rescue

## References

[1] Stephanides SL, Vohra T. Injury patterns and first aid training among canyoneers. Wilderness Environ Med. 2007;18(1):16–9. 17447708 10.1580/1080-6032(2007)18[16:ipafat]2.0.co;2

[2] Strapazzon G, Larsen G. Canyoneering and Canyon Medicine. In: Auerbach’s Wilderness Medicine. Elsevier, Philadelphia, PA, USA; pp. 2219–38.

[3] Soteras I, Subirats E, Strapazzon G. Epidemiological and medical aspects of canyoning rescue operations. Injury. 2015;46(4):585–9. 25640589 10.1016/j.injury.2014.12.030

[4] Steinman AM, Hayward JS, Nemiroff MJ, Kubilis PS. Immersion hypothermia: comparative protection of anti-exposure garments in calm versus rough seas. Aviat Space Environ Med. 1987;58(6):550–8. 3606516

[5] Jones DM, Weller RS, McClintock R, Roberts N, Zheng W, Dunn TL. Prevalence of hypothermia and critical hand temperatures during military cold water immersion training. Int J Circumpolar Health. 2023;82(1):2236777. 10.1080/22423982.2023.2236777 PMC10361000 37469312

[6] Falla M, Micarelli A, Hüfner K, Strapazzon G. The effect of cold exposure on cognitive performance in healthy adults: a systematic review. Int J Environ Res Public Health. 2021;18(18):9725. 34574649 10.3390/ijerph18189725 PMC8470111

[7] Strapazzon G, Reisten O, Argenone F, Zafren K, Zen-Ruffinen G, Larsen GL, et al. International commission for mountain emergency medicine consensus guidelines for on-site management and transport of patients in canyoning incidents. Wilderness Environ Med. 2018;29(2):252–65. 29422373 10.1016/j.wem.2017.12.002

[8] Darioli V, Taffé P, Carron PN, et al. Evaluation of the discriminative performance of the prehospital National Advisory Committee for Aeronautics score regarding 48-h mortality. Eur J Emerg Med. 2019;26(5):366–72. 30308574 10.1097/MEJ.0000000000000578

[9] Raatiniemi L, Mikkelsen K, Fredriksen K, Wisborg T. Do pre-hospital anaesthesiologists reliably predict mortality using the NACA severity score? A retrospective cohort study. Acta Anaesthesiol Scand. 2013;57(10):1253–9. 24134443 10.1111/aas.12208 PMC4287201

[10] Fiore PI, Monteleone AS, Jochen Müller, Filardo G, Candrian C, Riegger M. The NACA score predicts mortality in polytrauma patients before hospital admission: a registry-based study. Scand J Trauma Resusc Emerg Med. 2024;32(1):116. 10.1186/s13049-024-01281-3 PMC11575110 39558381

[11] Milani M, Roveri G, Falla M, Dal Cappello T, Strapazzon G. Occupational accidents among search and rescue providers during mountain rescue operations and training events. Ann Emerg Med. 2023;81(6):699–705. 36669910 10.1016/j.annemergmed.2022.12.015

[12] Ströhle M, Beeretz I, Rugg C, Woyke S, Rauch S, Paal P. Canyoning accidents in Austria from 2005 to 2018. Int J Environ Res Public Health. 2019;17(1):102. 31877835 10.3390/ijerph17010102 PMC6982325

[13] Rauch S, Dal Cappello T, Strapazzon G, et al. Pre-hospital times and clinical characteristics of severe trauma patients: a comparison between mountain and urban/suburban areas. Am J Emerg Med. 2018;36(10):1749–53. 29395773 10.1016/j.ajem.2018.01.068

[14] Davis CA, Schmidt AC, Sempsrott JR, et al. Wilderness medical society clinical practice guidelines for the treatment and prevention of drowning: 2024 update. Wilderness Environ Med. 2024;35:94S-111S. 38379489 10.1177/10806032241227460

[15] Sempsrott J, Hawkins S, Cushing T. Submersion injuries and drowning. In: Auerbach’s Wilderness Medicine. Elsevier, Philadelphia, PA, USA. 2017; pp. 1530–1549.

[16] Ballesteros Pena S. Evaluación de la adherencia a las medidas de seguridad en la practica deportiva del barranquismo en la sierra de Guara (Huesca). Arch Med Deport. 2013;30:91–5.

[17] Paal P, Gordon L, Strapazzon G, et al. Accidental hypothermia-an update : The content of this review is endorsed by the International Commission for Mountain Emergency Medicine (ICAR MEDCOM). Scand J Trauma Resusc Emerg Med. 2016;24(1):111. 27633781 10.1186/s13049-016-0303-7 PMC5025630

[18] Tanaka M, Tokudome S. Accidental hypothermia and death from cold in urban areas. Int J Biometeorol. 1991;34(4):242–6. 2055665 10.1007/BF01041837

[19] Barsten TW, Sunde E, Thomassen Ø, Mydske S. Methods and equipment available for prehospital treatment of accidental hypothermia: a survey of Norwegian prehospital services. Scand J Trauma Resusc Emerg Med. 2024;32(1):131. 39695744 10.1186/s13049-024-01302-1 PMC11653919

[20] Van Veelen MJ, Brodmann Maeder M. Hypothermia in trauma. Int J Environ Res Public Health. 2021;18(16):8719. 34444466 10.3390/ijerph18168719 PMC8391853

[21] Sumann G, Moens D, Brink B, et al. Multiple trauma management in mountain environments - a scoping review : Evidence based guidelines of the International Commission for Mountain Emergency Medicine (ICAR MedCom). Intended for physicians and other advanced life support personnel. Scand J Trauma Resusc Emerg Med. 2020;28(1):117. 33317595 10.1186/s13049-020-00790-1 PMC7737289

[22] Shafi S, Elliott AC, Gentilello L. Is hypothermia simply a marker of shock and injury severity or an independent risk factor for mortality in trauma patients? Analysis of a large national trauma registry. J Trauma. 2005;59(5):1081–5. 16385283 10.1097/01.ta.0000188647.03665.fd

[23] Pietsch U, Strapazzon G, Ambühl D, Lischke V, Rauch S, Knapp J. Challenges of helicopter mountain rescue missions by human external cargo: need for physicians onsite and comprehensive training. Scand J Trauma Resusc Emerg Med. 2019;27(1):17. 30760298 10.1186/s13049-019-0598-2 PMC6374883

