# Correction: The epidemiology of canyoning rescue operations in Italy: a retrospective 15-year database study

**DOI:** 10.1186/s13049-026-01660-y

**Published:** 2026-07-21

**Authors:** Alexandre Tomasi, Michiel Jan Van Veelen, Marco Biasioni, Tomas Dal Cappello, Giacomo Strapazzon

**Affiliations:** 1 Institute of Mountain Emergency Medicine, Eurac Research, Bolzano, Italy; 2 Corpo Nazionale Soccorso Alpino e Speleologico - CNSAS, Milano, Italy; 3 Department of Sport Science, Medical Section, University of Innsbruck, Innsbruck, Austria; 4 Department of Medicine – DIMED, University of Padova, Padova, Italy; 5 Italian Society of Mountain Medicine – SIMeM, Padova, Italy

**Correction: Scandinavian Journal of Trauma, Resuscitation and Emergency Medicine (2026) 34:31**

10.1186/s13049-025-01535-8

In this article the author’s name Tomas Dal Cappello was incorrectly written as Dal Tomas Cappello.

The original article has been corrected.

